# Nucleobindin‐2/nesfatin‐1 enhances the cell proliferation, migration, invasion and epithelial‐mesenchymal transition in gastric carcinoma

**DOI:** 10.1111/jcmm.17522

**Published:** 2022-09-06

**Authors:** Le Ren, Deming Bao, Liming Wang, Qin Xu, Yayun Xu, Zhenwang Shi

**Affiliations:** ^1^ Department of Gastroenterology Second People's Hospital of Hefei Hefei China; ^2^ Inflammation and Immune Mediated Diseases Laboratory of Anhui Province, School of Pharmacy Anhui Institute of Innovative Drugs, Anhui Medical University Hefei China

**Keywords:** gastric carcinoma, invasion, migration, NUCB2/nesfatin‐1, proliferation

## Abstract

Nesfatin‐1, a newly discovered adipokine derived from nucleobindin‐2 (NUCB2), has been described as a new prognostic marker in cancers. This study aimed to explore the functional role of NUCB2/nesfatin‐1 in the cell proliferation, migration and invasion in gastric carcinoma (GC). The expressions of NUCB2/nesfatin‐1 in GC tissues and normal adjacent tissues (NATs) were compared, and the effect of inhibition of NUCB2/nesfatin‐1 on the cell proliferation, migration, invasion and epithelial‐mesenchymal transition (EMT) in GC cell line SGC‐7901 was investigated. Cell transfection was conducted to inhibit NUCB2/nesfatin‐1 by short hairpin RNA. Cell proliferation, migration and invasion abilities were determined using cell counting kit‐8 (CCK‐8), 5‐ethynyl‐2′‐deoxyuridine (EdU), wound healing and transwell assays, respectively. The expressions of EMT markers E‐Cadherin and N‐Cadherin were determined using western blotting. The expression of NUCB2/nesfatin‐1 protein in GC tissues was significantly increased compared with that in NATs. Consistently, the serum concentrations of NUCB2/nesfatin‐1 were significantly higher in patients with GC as compared with those in the control group. Moreover, the results of CCK‐8 assay and EdU assay indicated that knockdown of NUCB2/nesfatin‐1 could markedly decrease SGC‐7901 proliferation. Furthermore, the results of wound healing assay and transwell assay demonstrated that knockdown of NUCB2/nesfatin‐1 significantly suppressed SGC‐7901 migration and invasion abilities. Additionally, knockdown of NUCB2/nesfatin‐1 decreased the expressions of N‐Cadherin and increased the expressions of E‐Cadherin in SGC‐7901 cells. These findings suggest that knockdown of NUCB2/nesfatin‐1 suppressed the proliferation, migration, invasion and EMT of SGC‐7901 cells, suggesting a potentially promising therapeutic target for GC.

## 1 INTRODUCTION

Gastric carcinoma (GC), one of the most common malignant tumours, is considered the second leading cause of cancer‐related deaths across the worldwide.^1^ Despite advances in diagnostic methods and the implementation of novel targeted therapies, the overall 5‐year survival rate for patients with GC remains disappointing.^2^ Previous studies have shown that the long‐term survival rate of patients with early‐stage GC was improved due to the advances in surgical techniques, adjuvant chemotherapy, radiotherapy and immune therapy; however, the prognosis of patients with late‐stage GC remains poor due to cancer invasion and metastasis.^3^, ^4^ Therefore, it is imperative to make great effort to investigate the molecular mechanism underlying GC invasion and develop novel therapeutic strategies to inhibit GC metastasis.

Nesfatin‐1, a recently discovered anorectic peptide encoded in the precursor protein nucleobindin‐2 (NUCB2),^5^ plays a role in appetite regulation through hypothalamic leptin‐independent mechanisms.^6^, ^7^ Emerging evidence has demonstrated close links between NUCB2/nesfatin‐1 and tumorigenesis.^8^ It has been reported that NUCB2/nesfatin‐1 could promote cell proliferation, migration and invasion in various cancers including glioblastoma,^9^ colon,^10^ endometrial,^11^ thyroid^12^ and bladder cancers.^13^ In terms of GC, previous study has shown that the peripheral NUCB2/nesfatin‐1 concentrations were significantly increased in patients with GC.^14^ Moreover, a high expression of NUCB2/nesfatin‐1 in GC tissues is significantly associated with tumour depth, lymph node metastasis, lymphatic invasion, venous invasion and clinical stage.^15^ However, to the best of our knowledge, the effect of NUCB2/nesfatin‐1 on the cell proliferation, migration and invasion in GC has not been explored.

Considering that NUCB2/nesfatin‐1 is an important factor in cancer development and progression, the aim of the present study was to investigate whether NUCB2/nesfatin‐1 was involved in the cell proliferation, migration and invasion in GC. To test this hypothesis, the expressions of NUCB2/nesfatin‐1 in GC tissues and normal adjacent tissues (NATs) were compared, and the effect of inhibition of NUCB2/nesfatin‐1 on the cell proliferation, migration, invasion and epithelial‐mesenchymal transition (EMT) in GC cell line SGC‐7901 was investigated.

## 2 MATERIALS AND METHODS

### 2.1 Patients

A total of 34 patients with GC, who were admitted to Second People's Hospital of Hefei, were enrolled into the present study between January 2019 and December 2021. All patients were diagnosed with GC according to pathological examination results. Thirty healthy individuals were selected as the control group within the same time period. Clinical information was obtained from the clinical records of the subjects. Patients with other types of cancer or major organ diseases, severe active infectious diseases, severe blood diseases, bone marrow transplantation, severe trauma or immune diseases were excluded. The present study was approved by the Ethics Committee of The Second People's Hospital of Hefei. Written informed consent for the use of samples and clinical data were obtained from all patients and healthy subjects.

### 2.2 Collection and measurement of tissue and serum samples

Samples of GC were collected from the GC patients who underwent gastrectomy at Second People's Hospital of Hefei and fixed in 10% buffered formalin. Blood samples from a forearm vein were drawn with the subjects in a fasting state. Serum was separated from whole blood in serum collection tubes by centrifugation at 1, 200 × *g* for 5 min at 4°C and stored at −80°C until analysis. Commercially available Enzyme‐Linked Immunosorbent Assay (ELISA) kit was used to measure the serum concentrations of NUCB2/nesfatin‐1 (Jianglai Bio) according to the manufacturer's instructions.

### 2.3 Immunochemical staining

The tissue samples were embedded in paraffin and cut into 4‐μm sections. Immunohistochemical staining of NUCB2/nesfatin‐1 in GC tissues was performed with the SP‐9000 Histostain‐Plus kits (ZSGB Bio) according to the manufacturer's instructions. Anti‐human NUCB2/nesfatin‐1 monoclonal antibody (Catalogue # MAB5949) was obtained from R&D Systems. Samples were visualized by a digital pathology slide scanner (3DHISTECH, The Digital Pathology Company, Budapest, Hungary). Image‐Pro Plus Software (Media Cybernetics) was used to calculate the integral optical density (IOD) of the sections.

### 2.4 Human gastric carcinoma cell lines and transfection

Human GC cell line SGC‐7901 was grown in Dulbecco's Modified Eagle Medium (DMEM) supplemented with 10% foetal bovine serum (Gibco BRL) and streptomycin and penicillin (100 U/ml). Adenoviral constructs carrying shRNA against *NUCB2* mRNA and control shRNA (negative control) were constructed by GenePharma Co. (Shanghai, China). The shRNA sequences against *NUCB2* mRNA used in this study were as follows: shRNA‐nesfatin‐1#1, AAGCTGTGCCTATTGACATAGAC; shRNA‐nesfatin‐1#2, AAGCAAAGAACTGGATTTAGTAA. For transfection, shRNA‐nesfatin‐1 NC, shRNA‐nesfatin‐1#1 and shRNA‐nesfatin‐1#2 were transfected into SGC‐7901 cells using Lipofectamine 3000 as the transfection reagent (Life Technologies, Inc.). At 48 h post‐transfection, the cells were harvested and quantitative real‐time reverse transcription PCR (RT‐qPCR) was conducted to determine the transfection efficiency.

### 2.5 qRT‐PCR assay

Total RNA was isolated from SGC‐7901 cells using TRIzol reagent (Invitrogen) and reversely transcribed to synthesize cDNA using a First Strand cDNA Synthesis Kit (Thermo Fisher Scientific). The qRT‐PCR reactions were performed using a CFX96 Real‐time RT‐PCR detection system (Bio‐Rad, USA) and a SYBR Premix Ex Taq kit (TaKaRa Biotechnology) under the following conditions: 30 s at 95°C, followed by 40 cycles of amplification (15 s at 95°C, 60 s at 62°C and 30 s at 72°C). The Ct values of the samples were calculated and the transcript levels were analysed using the 2^−ΔΔC*t*^ method. The sequences of primers are as follows: NUCB2/nesfatin‐1: (forward) 5′‐AAAGAAGAGCTACAACGTCA‐3′ and (reverse) 5′‐GTGGCTCAAACTTCAATTC‐3′; GAPDH: (forward) 5′‐GGAAAGCTGTGGCGTGAT‐3′ and (reverse) 5′‐AAGGTGGAAGAATGGGAGTT‐3′.

### 2.6 Cell counting kit‐8 (CCK‐8) assay

CCK‐8 (Dojindo) was chosen to detect cell proliferative capacity of SGC‐7901 cells. SGC‐7901 cells were seeded in 96‐well plates at 5 × 10^3^ cells per well. Each well was treated with 10 μl of CCK‐8 reagent, followed by incubation at 37°C for 1 h in the dark. Then, the absorbance at 450 nm of each well was measured using a microplate reader (Bio‐Rad). Cell proliferation was observed at different times (6, 24, 48, 72 and 96 h).

### 2.7 5‐Ethynyl‐20‐deoxyuridine (EdU) assay

To evaluate the proliferation viability of SGC‐7901 cells, the EdU assay was conducted with a BeyoClick™ EdU‐555 detection kits (Beyotime). Transfected RASFs were seeded in six‐well plates and incubated with complete medium for 24 h. After incubation with 50 mM EdU for 6 h, the cells were fixed and stained for 30 min. The nucleic acid was stained with Hoechst 33342. All images were captured with a fluorescent microscope.

### 2.8 Wound healing assay

For wound healing assay, SGC‐7901 cells were seeded in six‐well plates. When the cell confluence reached 90–100%, a sterile 200 μl pipette tip was used to make a straight scratch line on the monolayer of confluent cells at the bottom of the culture plate. Cell migration images were captured at time points of 0 h, 24 h and 48 h by an inverted microscope (Olympus Optical Co., Ltd.). Migrated distance was measured and quantified.

### 2.9 Transwell assays

For transwell assays, SGC‐7901 cells were seeded into the upper chamber that was pre‐treated with or without Matrigel (Matrigel BD biosciences) and each chamber loaded in 200 μl serum‐free culture medium and placed in 24‐well tissue culture dishes. The lower chambers were filled with 800 μl DMEM containing 20% FBS. After 24 h of incubation, upper chamber cells were removed and invaded cells were fixed and stained. All images were captured with microscope (Olympus).

### 2.10 Western blotting

Total protein was harvested using RIPA lysis buffer and PMSF (Beyotime Institute of Biotechnology) for 30 min and subsequently subjected to centrifugation at 12,000 rpm for 30 min at 4°C. The protein concentration was determined using the Pierce BCA Protein Assay Kit (Pierce), and equal amounts of protein were separated on 10% SDS‐polyacrylamide gels and transferred to polyvinylidene difluoride (PVDF) membranes (Millipore). Then, the membranes were blocked in Tris‐buffered saline with 0.1% Tween‐20 (TBST) supplemented with 5% skim milk and incubated with primary antibodies against E‐Cadherin (Catalogue # ab40772; Abcam) and N‐Cadherin (Catalogue # ab76011; Abcam) at 4°C overnight. Next, the membranes were incubated with HRP‐conjugated secondary antibody at room temperature for 2 h after washing TBST for 3 times. Protein bands were visualized using an enhanced chemiluminescence (ECL) kit (Millipore), and the band intensity was analysed using ImageJ software (National Institutes of Health).

### 2.11 Statistical analysis

Statistical data were analysed using SPSS (version 17.0; IBM Corp.). Data are expressed as the mean ± standard error of the mean (SEM) with *p* < 0.05 considered statistically significant. The cell viability data of CCK‐8 assay were analysed using repeated‐measures analysis of variance (anova) followed by the least significant difference (LSD) test. Student's *t* test was used for comparing means between two groups and one‐way anova with LSD post hoc test was used for comparisons among three or more groups, where appropriate. Correlation analyses were performed using Pearson correlation tests. The receiver operating characteristic (ROC) curve analysis was plotted to evaluate the area under the curve (AUC) of serum NUCB2/nesfatin‐1 level for diagnosing GC.

## 3 RESULTS

### 3.1 Upregulated levels of NUCB2/nesfatin‐1 in GC tissue and serum of patients with GC

**FIGURE 1:**
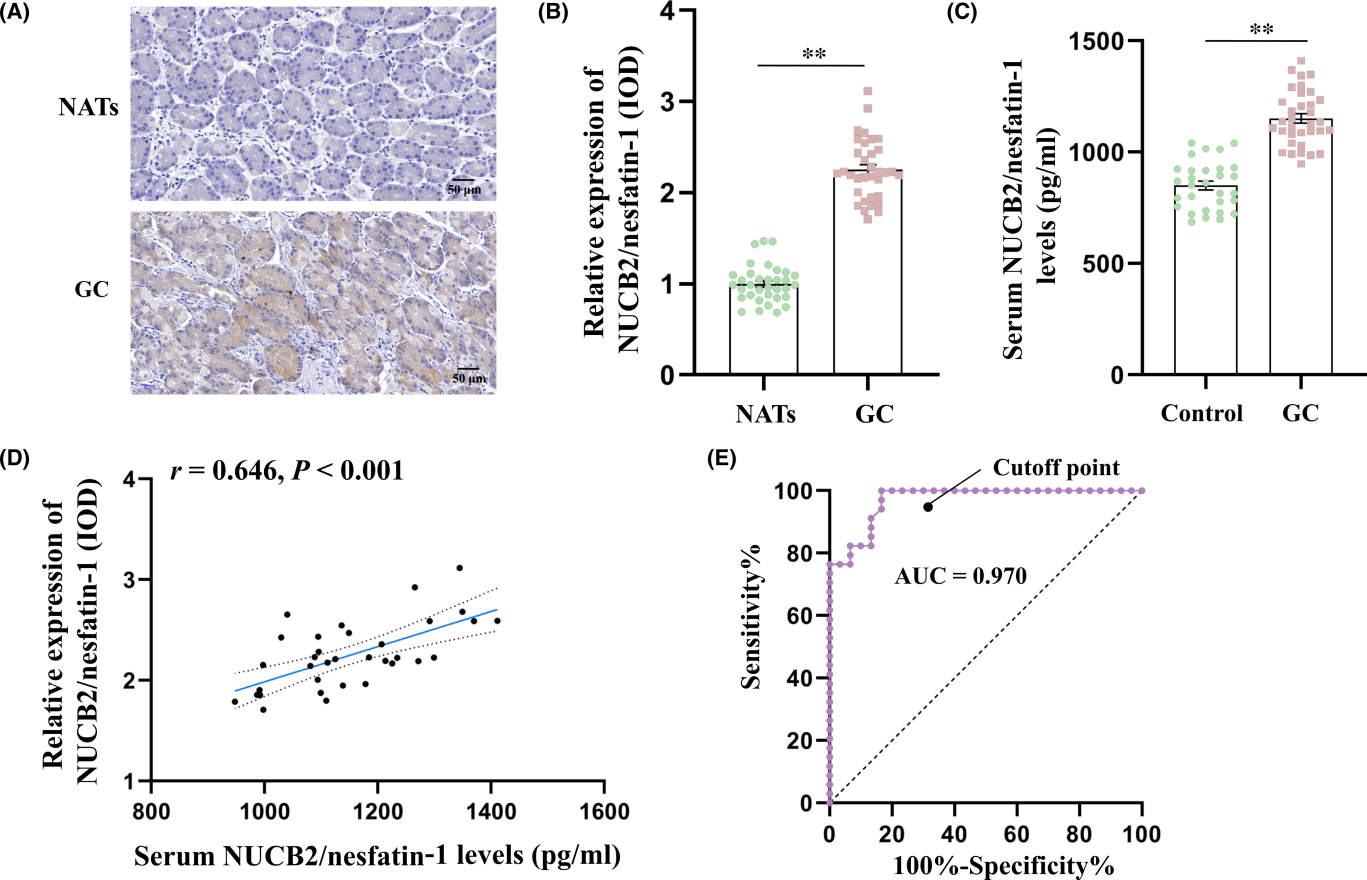
NUCB2/nesfatin‐1 expression in GC tissue and serum of patients with GC. (A) Typical immunohistochemistry images of NUCB2/nesfatin‐1 protein expression in GC tissues and NATs; (B) Quantitative analysis of NUCB2/nesfatin‐1 protein expression based on immunohistochemistry results; (C) Comparison of mean values of serum NUCB2/nesfatin‐1 in the GC and control groups; (D) Correlation between the serum levels of NUCB2/nesfatin‐1 and the relative protein expression of NUCB2/nesfatin‐1 (IOD) in GC tissues; (E) ROC curve of NUCB2/nesfatin‐1 in serum in identification of the patients with GC. The data are presented as the mean ± SEM. **p < 0.01 vs. control group or NATs

As shown in Figure 1A,B, the expression of NUCB2/nesfatin‐1 was localized in the cytoplasm of GC cells, and the expression of NUCB2/nesfatin‐1 protein in GC tissues was significantly increased compared with that in NATs (*t* = −18.704, *p* < 0.001; Figure 1A,B). Consistently, the serum concentrations of NUCB2/nesfatin‐1 in patients with GC were significantly higher as compared with those in the control group (*t* = −6.876, *p* < 0.001; Figure 1C). The serum levels of NUCB2/nesfatin‐1 were positively correlated to the relative protein expression of NUCB2/nesfatin‐1 (IOD) in GS tissues in patients with GC (*r* = 0.646, *p* < 0.001; Figure 1D). ROC curve analysis showed the potential diagnostic value of serum NUCB2/nesfatin‐1 for GC (Figure 1E). The AUC for NUCB2/nesfatin‐1 was 0.970 (95% confidence interval, 0.932–1.000). Furthermore, at a cut‐off NUCB2/nesfatin‐1 value of 939.07 pg/ml, the optimal sensitivity and specificity were 100% and 83.3%, respectively, in discriminating patients with GC from healthy controls.

### 3.2 Knockdown of NUCB2/nesfatin‐1 inhibited the proliferation in SGC‐7901 cells

**FIGURE 2:**
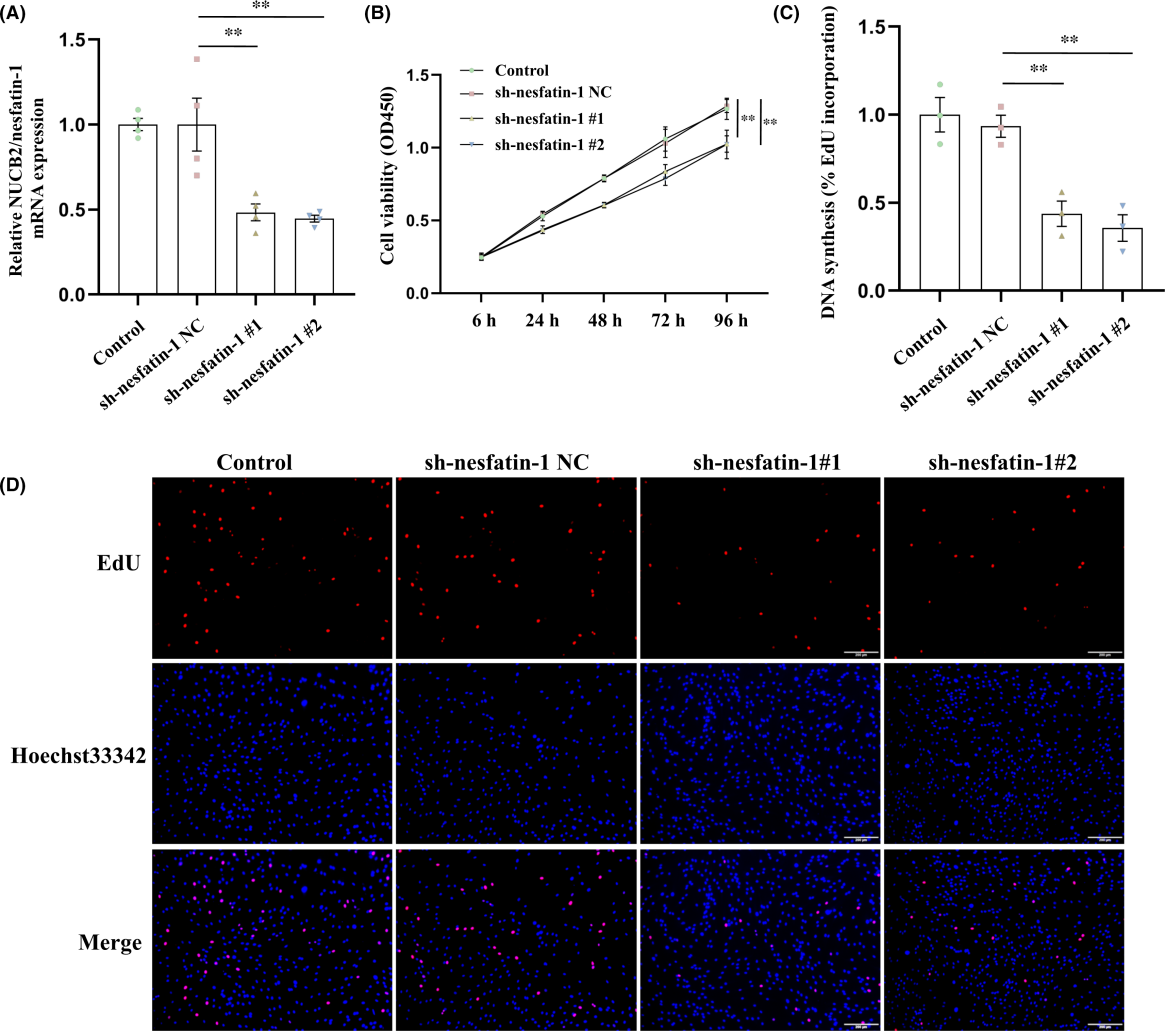
Effect of NUCB2/nesfatin‐1 knockdown on the proliferation of SGC‐7901 cells. (A) Interference efficiency of sh‐nesfatin‐1 NC, sh‐nesfatin‐1#1 and sh‐nesfatin‐1#2; (B) CCK‐8 assays were performed to detect cell proliferation; (C and D) EdU assays was performed to evaluate cell proliferation. All data are presented as the means ± SEM of three independent experiments. **p < 0.01 vs. sh‐nesfatin‐1 NC group

To investigate the function of NUCB2/nesfatin‐1 in GC, SGC‐7901 cells were stably transfected with sh‐nesfatin‐1 NC, sh‐nesfatin‐1#1 and sh‐nesfatin‐1#2. The results of qRT‐PCR assay showed that sh‐nesfatin‐1#1 and sh‐nesfatin‐1#2 could significantly decrease the mRNA expression of NUCB2/nesfatin‐1 compared with sh‐nesfatin‐1 NC (Figure 2A). CCK‐8 assay showed that cell proliferation ability in SGC‐7901 cells was significantly inhibited by knockdown of NUCB2/nesfatin‐1 (Figure 2B). Consistently, the results of EdU assay also showed that NUCB2/nesfatin‐1 knockdown significantly inhibited the proliferation of SGC‐7901 cells (Figure 2C,D).

### 3.3 Knockdown of NUCB2/nesfatin‐1 decreased the migration and invasion abilities in SGC‐7901 cells

**FIGURE 3:**
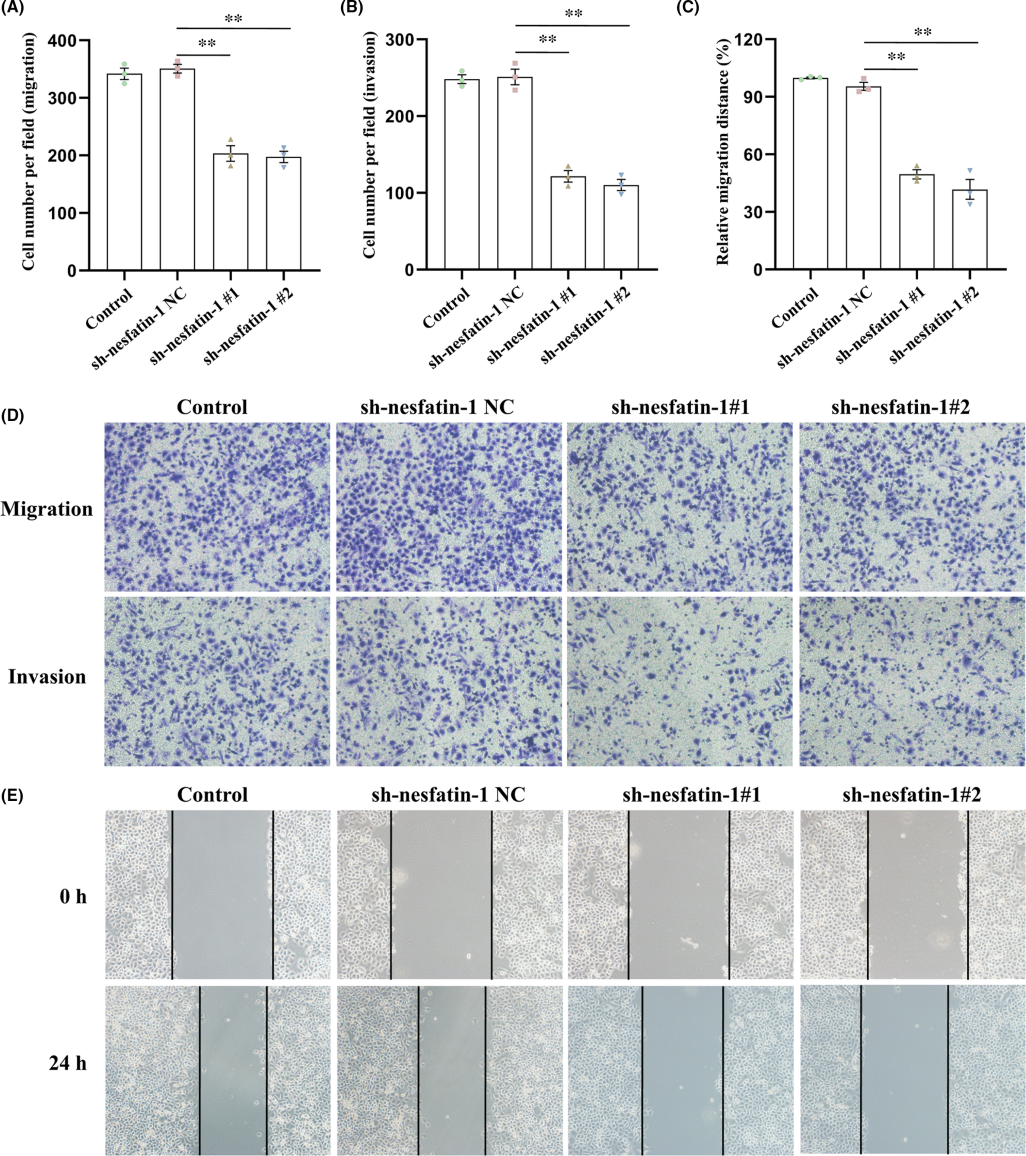
Effect of NUCB2/nesfatin‐1 knockdown on the migration and invasion of SGC‐7901 cells. (A,B and D) Transwell assays were performed to assess the migration and invasion abilities. The samples were imaged at 100× magnification; (C and E) Cell migration was assessed using a wound healing assay. The samples were imaged at 100× magnification. All data are presented as the means ± SEM of three independent experiments. **p < 0.01 vs. sh‐nesfatin‐1 NC group

As shown in Figure 3A,B,D, NUCB2/nesfatin‐1 knockdown in SGC‐7901 cells significantly inhibited cell migration and invasion, as assessed by a transwell migration assay (without Matrigel) and a transwell invasion assay (with Matrigel), respectively. Consistently, wound healing assays revealed that NUCB2/nesfatin‐1 knockdown in SGC‐7901 cells moderately decreased the migration rate (Figure 3C,E).

### 3.4 Knockdown of NUCB2/nesfatin‐1 decreased the expressions of N‐Cadherin and increased the expressions of E‐Cadherin in SGC‐7901 cells

**FIGURE 4:**
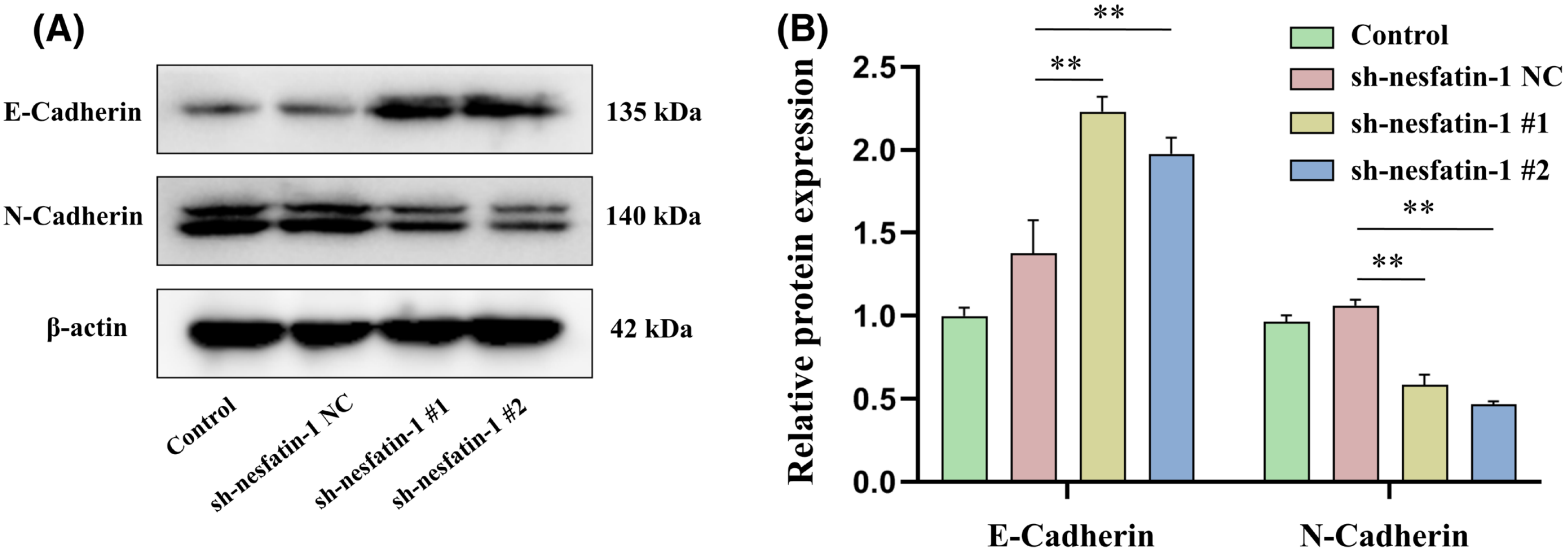
Effect of NUCB2/nesfatin‐1 knockdown on the E‐Cadherin and N‐Cadherin protein expression of SGC‐7901 cells. (A) Typical images of E‐Cadherin and N‐Cadherin protein expression; (B) Quantitative analysis of protein expression based on western blotting results. All data are presented as the means ± SEM of three independent experiments. **p < 0.01 vs. sh‐nesfatin‐1 NC group

Figure 4 showed the effect of NUCB2/nesfatin‐1 knockdown on the expressions of EMT markers E‐Cadherin and N‐Cadherin in SGC‐7901 cells. The expression of N‐Cadherin was significantly decreased, while the expression of E‐Cadherin was significantly increased in SGC‐7901 cells transferred with sh‐nesfatin‐1#1 or sh‐nesfatin‐1#2.

## 4 DISCUSSION

In the present study, we demonstrated that the expression of NUCB2/nesfatin‐1 protein in GC tissues was significantly higher compared with that in NATs. Consistently, the serum concentrations of NUCB2/nesfatin‐1 were significantly higher in patients with GC. Moreover, the results of CCK‐8 assay and EdU assay indicated that knockdown of NUCB2/nesfatin‐1 could markedly decrease SGC‐7901 proliferation. Furthermore, the results of wound healing assay and transwell assay demonstrated that knockdown of NUCB2/nesfatin‐1 significantly suppressed SGC‐7901 migration and invasion abilities. Additionally, knockdown of NUCB2/nesfatin‐1 decreased the expressions of N‐Cadherin and increased the expressions of E‐Cadherin in SGC‐7901 cells. These findings suggest that knockdown of NUCB2/nesfatin‐1 suppressed the proliferation, migration, invasion and EMT of SGC‐7901 cells.

Previous studies have suggested that the plasma level of NUCB2/nesfatin‐1 in patients of GC was significantly higher than that in healthy controls.^14^ Consistently, in the present study, the serum concentrations of NUCB2/nesfatin‐1 were also significantly higher in patients with GC, indicating that blood NUCB2/nesfatin‐1 level may serve as a novel biomarker for the diagnosis of GC. Therefore, ROC analysis was carried out to assess the potential value of serum NUCB2/nesfatin‐1 for GC diagnosis. The results showed that the serum NUCB2/nesfatin‐1 cut‐off point of 939.07 pg/mL showed a 100% sensitivity and a 83.3% specificity, indicating that serum NUCB2/nesfatin‐1 has a superior diagnostic value in GC. Given that early diagnosis is beneficial and critical for successful surgical resection of gastric cancer and greatly reduces the effectiveness of surgical interventions,^16^, ^17^ it is necessary to collect more blood samples from patients with early GC to evaluate the diagnostic value of serum NUCB2/nesfatin‐1 level in early GC.

The expression of NUCB2/nesfatin‐1 was localized in the cytoplasm of GC cells in the present study. No expression was found in the GC cell nucleus or the tumour stroma. Several studies have explored the expression of NUCB2/nesfatin‐1 in various tumours. Consistent to our results, it has been reported that the expression of NUCB2/nesfatin‐1 was localized in the cytoplasm of breast cancer^8^ and colon cancer cells.^18^ On the contrary, it has been revealed that NUCB2/nesfatin‐1 was predominantly expressed in the nucleus of glioblastoma cells.^9^ A recent study have indicated that NUCB2/nesfatin‐1 was expressed in both the nucleus and cytoplasm of papillary thyroid cancer cells.^12^ These findings suggest that NUCB2/nesfatin‐1 are expressed in a tissue‐ and cell‐specific manner. It should be noticed that Altan et al. found that NUCB2/nesfatin‐1 in GC cells was predominantly localized in the nuclei,^15^ which is inconsistent to our results. The reason for this discrepancy is currently unclear and warrants further examination. For example, the expression of NUCB2/nesfatin‐1 protein can be detected in different stages of GC to investigate whether the expression site of NUCB2/nesfatin‐1 has dynamic changes in GC.

Accumulating evidence has indicated that NUCB2/nesfatin‐1 expression was upregulated in cancer tissues compared to the noncancerous tissues obtained from the same patient in various tumours including colon cancer,^10^ prostate cancer,^19^ endometrial cancer^11^ and papillary thyroid cancer.^12^ Similarly, our results showed that the expression of NUCB2/nesfatin‐1 protein in GC tissues was significantly higher compared with that in NATs. Taken together, the phenomenon of upregulated expression in various tumour tissues suggests that NUCB2/nesfatin‐1 may play a common role in the occurrence and development of tumours. Further research is needed to better understand the abnormal expression of NUCB2/nesfatin‐1 and utilize it as a potential target in cancer therapy.

NUCB2/nesfatin‐1 has been reported to be an independent predictor of progression‐free survival of GC.^12^ Specifically, there was a close relationship between NUCB2/nesfatin‐1 expression in GC tissues and unfavourable progression‐free survival and overall survival. Moreover, positive correlations were found between NUCB2/nesfatin‐1 expression in GC tissues and the tumour depth, lymph node metastasis, lymphatic invasion, venous invasion and clinical stage. Therefore, the ability of NUCB2/nesfatin‐1 to regulate tumour metastasis and invasion was investigated in the present study. The results showed that knockdown of NUCB2/nesfatin‐1 significantly suppressed the proliferation, migration and invasion of SGC‐7901 cells. Cell migration and invasion are the two key processes during cancer metastasis, leading to the spread of cancer cells from primary tumours, which is the leading cause of death in cancer patients.^20^, ^21^ Altogether, NUCB2/nesfatin‐1 might be a potential therapeutic target for the inhibition of GC invasion and metastasis. Consistently, a number of in vitro studies have shown that knockdown of NUCB2/nesfatin‐1 could inhibit the cell proliferation in breast cancer,^22^ bladder cancer,^23^ glioblastoma,^9^ endometrial cancer^11^ and thyroid cancer cell lines.^12^ Surprisingly, it has been reported that treatment of H295R adrenocortical cells^24^ or endometrial cancer cell line^25^ with recombinant NUCB2/nesfatin‐1 resulted in a decreased proliferative capacity of the cells. Collectively, it is rational to assume that the role of NUCB2/nesfatin‐1 in cancers is variable and tissue‐specific.

EMT is a process in which cancer cells lose their polarity and cell–cell adhesion, change to mesenchymal fibroblast‐like cells and, finally, confer metastatic properties upon cancer cells through enhancing migratory and invasive abilities.^26^, ^27^ During EMT, increased expression of the mesenchymal markers N‐cadherin and the downregulation of the epithelial marker E‐cadherin, a powerful suppressor of tumour cell invasion and metastasis, have been observed.^28^ In the present study, the results showed that knockdown of NUCB2/nesfatin‐1 decreased the expressions of N‐Cadherin and increased the expressions of E‐Cadherin in SGC‐7901 cells, suggesting that suppression of the EMT process by NUCB2/nesfatin‐1 knockdown may contribute to inhibition of GC cell migration and invasion.

There are two limitations to this study. Firstly, considering that GAPDH has been shown to be upregulated in many cancers and downregulated by chemotherapic drugs,^29^ another housekeeping gene should be selected in qRT‐PCR assay. Secondly, the lack of in vivo experiments to confirm the functional role of NUCB2/nesfatin‐1 in GC is a limitation of the present study.

In conclusion, we found that knockdown of NUCB2/nesfatin‐1 obviously suppressed the proliferation, migration and invasion abilities and EMT process of SGC‐7901 cells. Since the NUCB2/nesfatin‐1 receptor has not yet been discovered, additional in vivo and in vitro researches are recommended to further investigate the role of NUCB2/nesfatin‐1 in the pathogenesis of GC.

## AUTHOR CONTRIBUTIONS

**Le Ren:** Conceptualization (equal); data curation (equal); formal analysis (equal); investigation (equal); methodology (equal); software (equal); writing – original draft (equal). **Deming Bao:** Formal analysis (equal); investigation (equal); supervision (equal). **Liming Wang:** Investigation (equal); resources (equal); supervision (equal); validation (equal). **Qin Xu:** Project administration (equal); resources (equal); software (equal). **Yayun Xu:** Formal analysis (equal); methodology (equal); writing – review and editing (supporting). **Zhenwang Shi:** Formal analysis (equal); funding acquisition (equal); project administration (equal); supervision (equal); validation (equal); visualization (equal); writing – review and editing (equal).

## FUNDING INFORMATION

This work was supported by the Research projects of The Second People's Hospital of Hefei (grant number: 2019015).

## CONFLICT OF INTEREST

The authors declare no conflicts of interest.

## Data Availability

The data that support the findings of this study are available from the corresponding author upon reasonable request.

## References

[1] Siegel R , Miller K , Fuchs H , Jemal A . Cancer statistics, 2021. CA Cancer J Clin. 2021;71(1):7‐33. 33433946 10.3322/caac.21654

[2] Joshi S , Badgwell B . Current treatment and recent progress in gastric cancer. CA Cancer J Clin. 2021;71(3):264‐279. 33592120 10.3322/caac.21657 PMC9927927

[3] Sexton R , Al Hallak M , Diab M , Azmi A . Gastric cancer: a comprehensive review of current and future treatment strategies. Cancer Metastasis Rev. 2020;39(4):1179‐1203. 32894370 10.1007/s10555-020-09925-3 PMC7680370

[4] Lazăr D , Avram M , Romoșan I , Cornianu M , Tăban S , Goldiș A . Prognostic significance of tumor immune microenvironment and immunotherapy: novel insights and future perspectives in gastric cancer. World J Gastroenterol. 2018;24(32):3583‐3616. 30166856 10.3748/wjg.v24.i32.3583 PMC6113718

[5] Stengel A , Mori M , Taché Y . The role of nesfatin‐1 in the regulation of food intake and body weight: recent developments and future endeavors. Obes Rev. 2013;14(11):859‐870. 23980879 10.1111/obr.12063 PMC3810163

[6] Elmquist J , Coppari R , Balthasar N , Ichinose M , Lowell B . Identifying hypothalamic pathways controlling food intake, body weight, and glucose homeostasis. J Comp Neurol. 2005;493(1):63‐71. 16254991 10.1002/cne.20786

[7] Oh‐I S , Shimizu H , Satoh T , et al. Identification of nesfatin‐1 as a satiety molecule in the hypothalamus. Nature. 2006;443(7112):709‐712. 17036007 10.1038/nature05162

[8] Kmiecik A , Dzięgiel P , Podhorska‐Okołów M . Nucleobindin‐2/Nesfatin‐1‐a new cancer related molecule? Int J Mol Sci. 2021;22(15):8313. 34361082 10.3390/ijms22158313 PMC8348729

[9] Liu Q , Lv J , Liu J , Zhang X , Wang L . Nucleobindin‐2 promotes the growth and invasion of glioblastoma. Cancer Biother Radiopharm. 2019;34(9):581‐588. 31697592 10.1089/cbr.2019.2829

[10] Kan J , Yen M , Wang J , et al. Nesfatin‐1/nucleobindin‐2 enhances cell migration, invasion, and epithelial‐mesenchymal transition via LKB1/AMPK/TORC1/ZEB1 pathways in colon cancer. Oncotarget. 2016;7(21):31336‐31349. 27150059 10.18632/oncotarget.9140 PMC5058760

[11] Takagi K , Miki Y , Tanaka S , et al. Nucleobindin 2 (NUCB2) in human endometrial carcinoma: a potent prognostic factor associated with cell proliferation and migration. Endocr J. 2016;63(3):287‐299. 26842712 10.1507/endocrj.EJ15-0490

[12] Zhao J , Yun X , Ruan X , et al. High expression of NUCB2 promotes papillary thyroid cancer cells proliferation and invasion. Onco Targets Ther. 2019;12:1309‐1318. 30863097 10.2147/OTT.S184560 PMC6388962

[13] Liu G , Xu Z , Ma H . Nesfatin‐1/Nucleobindin‐2 is a potent prognostic marker and enhances cell proliferation, migration, and invasion in bladder cancer. Dis Markers. 2018;2018:4272064. 30327690 10.1155/2018/4272064 PMC6169241

[14] Wang X , Zheng Y , Fang P , Song X . Nesfatin‐1 is a potential diagnostic biomarker for gastric cancer. Oncol Lett. 2020;19(2):1577‐1583. 31966083 10.3892/ol.2019.11200 PMC6956107

[15] Altan B , Kaira K , Okada S , et al. High expression of nucleobindin 2 is associated with poor prognosis in gastric cancer. Tumour Biol. 2017;39(7):1010428317703817. 28714371 10.1177/1010428317703817

[16] Wu H , Lin W , Tsai K . Advances in molecular biomarkers for gastric cancer: miRNAs as emerging novel cancer markers. Expert Rev Mol Med. 2014;16:e1. 24456939 10.1017/erm.2013.16 PMC3908627

[17] Wang F , Shen L , Li J , et al. The Chinese Society of Clinical Oncology (CSCO): clinical guidelines for the diagnosis and treatment of gastric cancer. Cancer Commun (Lond). 2019;39(1):10. 30885279 10.1186/s40880-019-0349-9 PMC6423835

[18] Xie J , Chen L , Chen W . High NUCB2 expression level is associated with metastasis and may promote tumor progression in colorectal cancer. Oncol Lett. 2018;15(6):9188‐9194. 29805650 10.3892/ol.2018.8523 PMC5958760

[19] Zhang H , Qi C , Li L , Luo F , Xu Y . Clinical significance of NUCB2 mRNA expression in prostate cancer. J Exp Clin Cancer Res. 2013;32(1):56. 23958433 10.1186/1756-9966-32-56 PMC3751731

[20] Friedl P , Wolf K . Tumour‐cell invasion and migration: diversity and escape mechanisms. Nat Rev Cancer. 2003;3(5):362‐374. 12724734 10.1038/nrc1075

[21] Entschladen F , Drell T , Lang K , Joseph J , Zaenker K . Tumour‐cell migration, invasion, and metastasis: navigation by neurotransmitters. Lancet Oncol. 2004;5(4):254‐258. 15050959 10.1016/S1470-2045(04)01431-7

[22] Suzuki S , Takagi K , Miki Y , et al. Nucleobindin 2 in human breast carcinoma as a potent prognostic factor. Cancer Sci. 2012;103(1):136‐143. 21988594 10.1111/j.1349-7006.2011.02119.x PMC11164150

[23] Cho J , Moon K , Lee H , et al. Nucleobindin 2 expression is an independent prognostic factor for bladder cancer. Medicine. 2020;99(13):e19597. 32221080 10.1097/MD.0000000000019597 PMC7220399

[24] Ramanjaneya M , Tan B , Rucinski M , et al. Nesfatin‐1 inhibits proliferation and enhances apoptosis of human adrenocortical H295R cells. J Endocrinol. 2015;226(1):1‐11. 25869615 10.1530/JOE-14-0496

[25] Xu Y , Pang X , Dong M , Wen F , Zhang Y . Nesfatin‐1 inhibits ovarian epithelial carcinoma cell proliferation in vitro. Biochem Biophys Res Commun. 2013;440(4):467‐472. 23764403 10.1016/j.bbrc.2013.06.001

[26] Thompson E , Newgreen D , Tarin D . Carcinoma invasion and metastasis: a role for epithelial‐mesenchymal transition? Cancer Res. 2005;65(14):5991‐5995. discussion 5995. 16024595 10.1158/0008-5472.CAN-05-0616

[27] Ribatti D , Tamma R , Annese T . Epithelial‐mesenchymal transition in cancer: a historical overview. Transl Oncol. 2020;13(6):100773. 32334405 10.1016/j.tranon.2020.100773 PMC7182759

[28] Lamouille S , Xu J , Derynck R . Molecular mechanisms of epithelial‐mesenchymal transition. Nat Rev Mol Cell Biol. 2014;15(3):178‐196. 24556840 10.1038/nrm3758 PMC4240281

[29] Valenti M , Bertoldo F , Dalle Carbonare L , et al. The effect of bisphosphonates on gene expression: GAPDH as a housekeeping or a new target gene? BMC Cancer. 2006;6:49. 16515701 10.1186/1471-2407-6-49 PMC1473200

